# A 2-year follow-up study of patients with pharyngotonsillitis

**DOI:** 10.1186/s12879-017-2917-4

**Published:** 2018-01-02

**Authors:** Jon Pallon, Martin Sundqvist, Katarina Hedin

**Affiliations:** 1 Department of Clinical Sciences, Malmö, Family Medicine, Lund University, Lund, Sweden; 2 Department of Research and Development, Region Kronoberg, Box 1223, 351 12 Växjö, Sweden; 3 Faculty of Medicine and Health, Department of Laboratory Medicine, Clinical Microbiology, University Hospital, Örebro, Sweden; 4 Primary Care Unit of Research and Development, Region Jönköping County, Jönköping, Sweden

**Keywords:** Pharyngitis, Etiology, Primary healthcare, Fusobacterium necrophorum

## Abstract

**Background:**

Longtime follow-up studies on patients with pharyngotonsillitis are rare. We aimed to describe the patterns of new visits for a sore throat, complications and tonsillectomy during 2 years in a cohort of patients with pharyngotonsillitis and non-infected controls.

**Methods:**

A retrospective chart review was performed on a cohort of patients with acute sore throat (*n* = 207), and non-infected controls (*n* = 108). New visits, complications and tonsillectomy within 2 years was recorded and analyzed in relation to microbiological findings at inclusion.

**Results:**

Patients with Group A streptococci (GAS) (12/66) reconsulted more often within 30 days than patients with no GAS (9/141) (*p* = 0.009) and patients with *F. necrophorum* (2/29). After 2 years, we observed no significant differences in reconsultations with regard to aetiology at inclusion. A single complication was recorded and 5 patients were planned for tonsillectomy.

**Conclusions:**

Group A streptococci were the sole aetiological agent associated with recurrent sore throat while *F. necrophorum* did not distinguish itself as a major cause of either recurrent infection or complications in this cohort. More studies, preferably with the focus on adolescents, are needed before *F. necrophorum* can be considered an important cause of pharyngotonsillitis.

## Background

Acute pharyngotonsillitis constitutes one fifth of all visits for respiratory tract infections in Swedish primary healthcare [1]. The most common causative agent is *Streptococcus pyogenes* (Group A streptococcus, GAS) [2] but several other bacteria and viruses have also been associated with the condition [2, 3], among these Streptococcus group C and G, *Mycoplasma pneumoniae* and *Arcanobacterium haemolyticum*. Furthermore, *Fusobacterium necrophorum* has been suggested as a possible pathogen in tonsillitis [4–8] and reported to be the second most common bacterial finding [6]. However, no one has so far studied the course of these patients, and studies on the course of patients with pharyngotonsillitis where modern diagnostic approaches and treatment recommendations have been used are also lacking.

Pharyngotonsillitis is associated with short-term complications such as sinusitis, otitis and peritonsillar abscess in a small percentage of patients [9]. Historically, post-streptococcal acute rheumatic fever and glomerulonephritis were dreaded conditions, but these are now uncommon in industrialized countries [2]. In some cases, recurrent infections lead to tonsillectomy [10, 11], but the long-term complications of an episode of pharyngotonsillitis have very rarely been studied, especially in relation to the aetiology of the condition.

Recently, we performed a case-control study on the aetiology of pharyngotonsillitis in young Swedish adults with a special focus on the importance of *F. necrophorum* as a possible pathogen [6]. The present study is a follow-up on that study with the purpose of observing patients over a 2-year period after a pharyngotonsillitis episode together with a cohort of non-infected patients. Specifically, our objective was to quantify the proportion of patients who would have a new doctor’s appointment for a sore throat within 2 years; have a complication of pharyngotonsillitis within 30 days; undergo or be planned for tonsillectomy within 2 years. These outcomes were studied in relation to the identified microorganism at inclusion.

## Methods

As previously described [6], a prospective case-control study was performed in 5 primary healthcare centres in southern Sweden during 2 subsequent winter periods (October–March, 2010–12). Patients aged 15–45 years presenting with acute sore throat and assessed to be in need of seeing a physician according to Swedish guidelines [12], were asked to participate [6]. Samples were collected from throat, nasopharynx and blood and screened for 20 different viruses and bacteria, using either culture, PCR or serology [6]. The following microorganisms were analyzed: β-hemolytic streptococci (Lancefield group A, C, and G), *Fusobacterium necrophorum, Mycoplasma pneumoniae, Chlamydophila pneumoniae*, Epstein-Barr virus, Adenovirus, Bocavirus, Coronavirus NL63, Coronavirus OC43, Coronavirus HKU1, Coronavirus 229E, Enterovirus, Influenza A virus, Influenza B virus, Metapneumovirus, Parainfluenzavirus, Rhinovirus and Respiratory syncytial virus. Controls were recruited among patients aged 15–45 years who presented at the healthcare centre for any other reason than respiratory tract infections.

### Follow-up

For both the patient and the control cohort medical files were reviewed retrospectively for the 2 subsequent years following inclusion. Data was retrieved from the comprehensive countywide electronic medical record system that covers both general practice and hospital care (Cambio Cosmic, Cambio Healthcare Systems, Linköping, Sweden) in Kronoberg county, Sweden. A standardized protocol was constructed to facilitate the review. Information about new visits for a sore throat, complications within 30 days after inclusion and tonsillectomy was retrieved from routine records. Based on previous studies [9, 13], we defined a complication as one of the following conditions occurring within 30 days after inclusion: sinusitis, peritonsillitis, media otitis, cellulitis, meningitis, sepsis, glomerulonephritis or rheumatic fever. A new visit was defined as a new doctor’s visit in either primary or secondary healthcare with an acute sore throat as the main symptom, including both non-resolving cases and recurrence with a symptom-free interval. Surgery was defined as either tonsillectomy or tonsillotomy, or being planned for this after consultation with an otorhinolaryngologist.

To minimize documentation bias, entries were read through in full and assessed for possible re-labelling of ICD-10 codes for outcomes. The review was performed by the principle author (JP), who was blinded to study data from the inclusion. Ambiguous medical entries were discussed with KH. Both researchers are general practitioners. Study subjects leaving the county during the follow-up period were not reviewed but confirmed alive through the Swedish population register.

#### Statistical analysis

Protocol data was merged with inclusion data and transferred to SPSS 23.0 software (IBM, Armonk, NY, USA) for descriptive statistics and for two-sided χ^2^-testing of proportions of categorical variables. Where expected numbers were low, a two-sided Fisher’s exact test was used.

In accordance with Hedin et al. [6], the microorganisms were grouped as: no pathogen”,” only viruses”,” only bacteria”,” only GAS (*Streptococcus pyogenes*)”,” only *F. necrophorum*” and” only Influenza B″. Consequently, all groups were not mutually exclusive.

For calculations of new visits, we used “30 days” and “2 years” after inclusion as points in time.

Power estimation and sample sizing was primarily calculated by Hedin et al. [6] for the aetiological study, and not for the follow-up.

## Results

### Characteristics of patients and controls

**Fig. 1:**
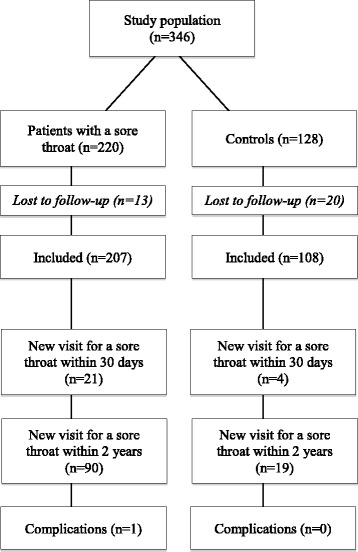
Two-year follow-up of patients with a sore throat in primary healthcare

All 220 patients and 128 controls originally included were confirmed alive at follow-up after 2 years. Thirteen patients and 18 controls had moved away from the county, one control was already included as a patient and yet another control was mistakenly registered twice, leaving 207 patients (94%) and 108 controls (86%) eligible for follow-up (Fig. 1). Median age was 34 among patients (range 15–48) and 33 among controls (range 16–46). Other characteristics of the 2 groups are presented in Table 1. Among the patients and controls lost to follow-up, the median age was 22 and 24, respectively (Table 2).

**Table 1:** Characteristics of the study population

	Percent		
	Patients (n = 207)	Controls (*n* = *108*)	χ^2^ *p*
Female	65	76	0.051
Smoker	14	8	0.090
History of often having a sore throat	34	6	<0.001
Previous tonsillectomy	14	13	0.70
Antibiotic treatment in the past month	7	3	0.11

**Table 2:** Characteristics of the missing cases in the follow-up of patients with sore throat in primary healthcare in relation to aetiology at inclusion

	Number		
	Patients	Female	Median age (yy)
All	13	6	22
No pathogen	5	4	21
Only viruses	3	0	29
Only bacteria	5	2	19
GAS	–	–	–
*F. necrophorum* (only)	4	2	19
Influenza B	–	–	–

### New visits for a sore throat

Of all patients 90/207 (43%) visited a doctor at least once for a sore throat during the 2-year follow-up period, compared to 19/108 (18%) in the control group (*p* < 0.001). At 30 days after inclusion, the corresponding proportions were 21/207 (10%) among patients and 4/108 (4%) among controls (*p* = 0.045). Of the 21 patients 4 had non-resolving symptoms and 17 presented with a new episode.

In the group with GAS as the sole microbiological finding at inclusion, 9/46 (20%) patients made a new visit within 30 days, which was significantly higher than among patients with no GAS (9/141 (6.4%); *p* = 0.018, Fisher’s). This difference remained even if the GAS group included the 20 additional patients where GAS was found together with other pathogens (12/66 (18%); *p* = 0.009).

None of the 10 patients with *F. necrophorum* as the only finding reconsulted for a sore throat within 30 days, in contrast to 19/178 (11%) of patients with no *F. necrophorum* (*p* = 0.08, Fisher’s). When considering all patients where *F. necrophorum* was found, either alone or together with other pathogens, 2/29 (7%) reconsulted within 30 days (*p* = 0.74, Fisher’s, compared to no *F. necrophorum*).

**Fig. 2:**
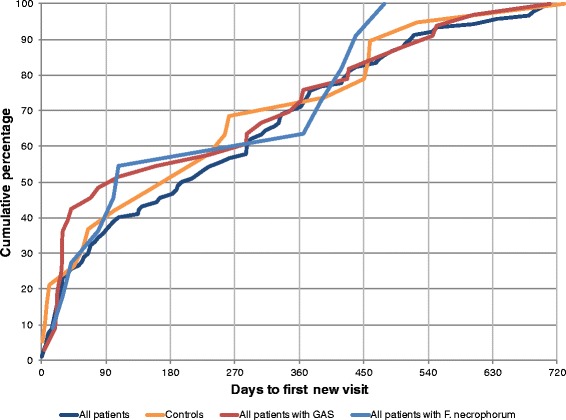
Time to first new visit for a sore throat. The graph illustrates the pattern of new visits for sore throat over time where the cumulative percentage of 100 corresponds to the total number of new visits in each patient group within 2 years from inclusion. The overall percentage of new visits were: 90/207 (43%) for “all patients”, 19/108 (18%) for “controls”, 33/66 (50%) for “any GAS” and 11/29 (38%) for “any *F. necrophorum*”. A “new visit” was only counted once for each patient

The differences observed at 30 days were less evident after 2 years, although the group with GAS as the only finding still had the highest proportion (52%) of at least one reconsultation. At this point, however, the differences were not statistically significant (Table 3). The temporal distribution of new visits is presented in Fig. 2, with separate cumulative percentages for “all patients”, “controls”, “all patients with GAS” and “all patients with *F. necrophorum*”.

**Table 3:** Proportion (%) of patients and controls attending for a sore throat within 30 days and 2 years, respectively, in relation to microbiological findings at inclusion

Microbiological finding at inclusion	Percent	
	30 days	2 years
Controls (*n* = 108)	4	18
Patients (*n* = 207)	10	43
No pathogen (*n* = 60)	7	38
Only viruses (*n* = 49)	8	47
Only bacteria (*n* = 80)	14	50
GAS only (*n* = 46)	20	52
* F. necrophorum* only (*n* = 10)	–	40
Influenza B only (*n* = 13)	–	46

### Complications and surgery

We excluded 2 patients from follow-up due to an ongoing complication (sinusitis and peritonsillitis) already at inclusion. In these two, no pathogen had been found. Among the remaining patients, 1 of 205 presented with sinusitis within 30 days, as compared to no complications in the control group. This patient had *F. necrophorum* as a single pathogen at inclusion. Among patients without previous tonsillar surgery, 5/178 (2.8%) either underwent or were planned for surgery during the follow-up period, as compared to none in the control group. In these 5 cases, the microorganisms at inclusion were the following: only viruses (*n* = 1), only *F. necrophorum* (n = 1), only GAS (n = 1) and no pathogen found (*n* = 2).

### Antibiotics

Of the 207 patients, 91 (44%) received an antibiotic prescription at inclusion. Antibiotics were prescribed more often to patients with a Centor score of 3–4 (56/80 (70%)) than to patients with Centor score 0–2 (35/127 (28%)) (*p* < 0.001). When comparing aetiological groups, patients with only GAS had the highest proportion of prescriptions: 44/46 (96%), followed by the group with only bacteria: 58/80 (73%), only *F. necrophorum*: 3/10 (30%), no pathogen found: 13/60 (22%) and only viruses: 7/49 (14%). The patient who developed sinusitis as a complication did not receive antibiotic treatment at inclusion.

No significant difference was seen between treated or untreated patients regarding new visits for a sore throat, either within 30 days or after 2 years. This observation held true both within the different aetiological groups and in the patient group as a whole (data not shown).

Further analysis of the group with only GAS based on Centor criteria, revealed that antibiotics were prescribed equally often irrespective of Centor score (Table 4).

**Table 4:** Proportion of patients with GAS only (*n* = 46) and new visits, in relation to Centor Score and antibiotics prescribed at first visit

	Percent (*n*)	
	30 d	2 years
Centor 0–2 (*n* = 15)	20 (3)	60 (9)
Antibiotics (*n* = 13)	15 (2)*	54 (7)**
Antibiotics (*n* = 2)	50 (1)	100 (2)
Centor 3–4 (*n* = 31)	19 (6)	48 (15)
Antibiotics (*n* = 31)	19 (6)	48 (15)
Antibiotics (*n* = 0)	–	–

*p = 0.37 (Fisher’s exact test), **p = 0.49 (Fisher’s exact test), when + “antibiotics” is compared to - “antibiotics”

## Discussion

In this study, we followed a well-described cohort of patients with pharyngotonsillitis and non-infected controls in primary healthcare for 2 years after inclusion, with special focus on the aetiology [6]. We observed a high tendency in patients to return with a sore throat within 2 years irrespective of microbiological finding at inclusion, while patients with GAS more often returned within 30 days as compared to patients with other possible aetiology of their disease. Only one complication was recorded (sinusitis) and 2.8% of the patients underwent tonsillectomy within 2 years after inclusion.

The main strength of this study is that it links the aetiological study on pharyngotonsillitis [6], where modern techniques were used, with both short- (30 days) and long-term (2-year) follow-up data.

The medical file review was carried out in a comprehensive electronic medical record system that covered both general practice and hospital care in the county. This increased the possibility to catch all relevant events. Possibly, a few patients may have sought medical advice outside the county.

The main weakness of this study, however, is its small size, being powered rather for the aetiological mapping than for prospective follow-up of uncommon events. This has increased the risk of missing true differences between groups, as well as it prevented from adjusting for confounders such as smoking, age, socioeconomic status and morbidity. Hedin et al. did, however, only find smoking and tonsillar coating to be associated with *F. necrophorum* at inclusion [6]. The rate of complications and surgery was also in line with previous reports [9].

Research on children has suggested that immediate prescription increases the risk of both relapse and recurrent infections [14], and Little et al. found that prescribing antibiotics lead to medicalisation and increased re-attendance in patients with sore throat [15]. In our study, the group of patients with only GAS had the highest proportion of reconsultations within 30 days. In Swedish primary care, rapid antigen detection tests for GAS are readily available, and one hypothesis could be that the mere identification of GAS changes the way physicians communicate with their patients. This may in turn affect the patients’ view on relapsing symptoms and hence lower their threshold for re-attendance. The fact that most patients with GAS were prescribed antibiotics, and equally often regardless of Centor score, could reflect both an excessive use of rapid antigen tests and GP’s making treatment decisions based on microbiological findings rather than clinical severity. The high prescription rate among patients with GAS and *F. necrophorum* (despite the physicians being unaware of the latter) may have reduced the number of complications observed in this study. However, a study on respiratory tract infections in general practice found that even a large reduction in antibiotic prescribing was only associated with a small increase in the number of complications [16].

While *F. necrophorum* was the second most prevalent pathogen in the aetiological study [6], it does not seem to compete with GAS aetiology regarding new visits in the short-term perspective. Rather, the patients with *F. necrophorum* were positioned with the groups with “only viruses” and “no pathogen” detected. However, the power of this result was somewhat diminished by 4 young patients with *F. necrophorum* leaving the county before follow-up.

As the proportion of patients with new visits evened out between groups over time, the aetiology did not seem to matter in the long perspective. This finding, together with the finding that the patients had more new visits than the controls, might suggest that a subset of the general population more often than the average experience a sore throat (as subjectively reported in the background characteristics) and/or have a lower threshold for attending medical care. The proportion of controls that re-attended was higher than we had anticipated. It must also be pointed out that a sore throat can have non-infectious causes, and that this study might have miss-classified some of the new visits as infectious.

According to current guidelines, the main reason for treating an acute sore throat with antibiotics is to alleviate symptoms in patients with more severe presentations, rather than preventing complications or surgery [2]. This study does not contradict these recommendations.

The significance of *F. necrophorum* in an acute sore throat has been debated: we saw previously that the bacterium was highly prevalent (15%) in the studied cohort, only outnumbered by GAS [6], and Centor found it to be even more common (20% prevalence) in a student population aged 15 to 30 [7]. Similarly, other researchers have identified *F. necrophorum* more often in patients than in controls [5, 8, 17] and Klug states that the role of *F. necrophorum* in acute tonsillitis seems significant but has to be clarified [18]. These studies were all focused on the acute illness and did not include a follow-up study. Jensen, however, analysed throat swabs retrospectively among patients aged 10 to 40 and found *F. necrophorum* in 11% of patients with acute non-streptococcal group A tonsillitis, but in 23% of patients with recurrent tonsillitis, which supports the view that the bacterium could be especially involved in such conditions [5]. Similarly, our group has found *F. necrophorum* to be common before tonsillectomy but also prevalent (16%) six months post-tonsillectomy, despite the fact that all these patients were then asymptomatic. This emphasizes the hypothesis that *F. necrophorum* may only cause a throat infection under certain circumstances [19]. In this study, we have not found any support for *F. necrophorum* as a more pathogenic finding than other pathogens in patients with pharyngotonsillitis with regard to new visits, complications or surgery within 2 years of infection.

## Conclusions

This study verifies that Group A Streptococci still is to be considered the most important pathogen in pharyngotonsillitis, associated with a higher number of new visits within 30 days, and that *F. necrophorum* did not distinguish itself as a major cause of recurrent infection or complications. These results do not merit any expansion of the aetiological paradigm of pharyngotonsillitis as suggested by others [20]. More studies, preferably treatment studies with the focus on the aetiology (and especially *F. necrophorum*) in adolescents with a sore throat, are needed before *F. necrophorum* can be confirmed or discarded as an important pathogen in pharyngotonsillitis.

## Abbreviations

GAS: Group A Streptococcus
